# Prevalence and comorbidity rates of disruptive mood dysregulation disorder in epidemiological and clinical samples: systematic review and meta-analysis

**DOI:** 10.1192/j.eurpsy.2024.1813

**Published:** 2025-01-15

**Authors:** Xavier Benarous, Hélène Lahaye, Angèle Consoli, David Cohen, Réal Labelle, Jean-Marc Guilé

**Affiliations:** 1 INSERM UMR-S 1136 IPLESP-ESSMA (Pierre Louis Institute of Epidemiology, Team Social Epidemiology, Mental Health, Addictions), Paris, France; 2 Department of Child and Adolescent Psychopathology, Amiens University Hospital, Amiens, France; 3 INSERM Unit U1105 Research Group for Analysis of the Multimodal Cerebral Function, University of Picardy Jules Verne (UPJV), Amiens, France; 4 Department of Child and Adolescent Psychiatry, Pitié-Salpêtrière Hospital, APHP.Sorbonne-Université, Paris, France; 5 CNRS UMR 7222, Institute for Intelligent Systems and Robotics, Sorbonne University, Paris, France; 6 Department of Psychology, Quebec University, Montreal, QC, Canada; 7 Centre for Research and Intervention on Suicide, Ethical Issues and End-of-Life Practices, Quebec University, Montreal, QC, Canada; 8 Department of Psychiatry, McGill University, Montreal, QC, Canada; 9 Department of Child and Adolescent Psychiatry, Etablissment Publique de Santé Mentale (EPSM) de la Somme, Amiens, France

**Keywords:** aggression, depressive disorder, emotional dysregulation, irritability, mood dysregulation, pediatric depression, temper outburst

## Abstract

**Background:**

This systematic review and meta-analysis evaluates the prevalence of disruptive mood dysregulation disorders (DMDD) in community-based and clinical populations.

**Methods:**

PubMed and PsychINFO databases were searched, using terms specific to DMDD, for studies of prevalence and comorbidity rates conducted in youths below 18.

**Results:**

Fourteen studies reporting data from 2013 to 2023 were included. The prevalence of DMDD in the community-based samples was 3.3% (95% confidence interval [CI], 1.4–6.0) and 21.9% (95% CI, 15.5–29.0) in the clinical population. The differences in the identification strategy of DMDD were associated with significant heterogeneity between studies in the community-based samples, with a prevalence of 0.82% (95% CI, 0.11–2.13) when all diagnosis criteria were considered. Anxiety, depressive disorders, and ADHD were the most frequent comorbidity present with DMDD. The association with other neurodevelopmental disorders remained poorly investigated.

**Conclusions:**

Caution is required when interpreting these findings, considering the quality of the reviewed data and the level of unexplained heterogeneity among studies. This review stresses the importance of considering a strict adhesion to DMDD criteria when exploring its clinical correlates.

## Introduction

Disruptive mood dysregulation disorder (DMDD) was introduced in the diagnostic and statistical manual of mental disorders, fifth edition (DSM-5), to characterize youths with chronic irritability associated with severe and recurrent episodes of temper outbursts [1]. This entity has been included within the depressive disorders section of the DSM-5 based on several lines of evidence from genetically informative, imaging, and longitudinal studies suggesting shared pathophysiological mechanisms among chronic irritability and depressive symptoms in childhood and adolescence [2–7].

Several studies have reported a higher level of functional impairment in children and adolescents with DMDD compared to those affected by other psychiatric disorders [8, 9]. Youths with DMDD seem particularly affected in the academic domain, with a high level of documented learning difficulties, grade repetition, school suspension, and relational difficulties with peers [10, 11]. Other lines of evidence showed that adverse effects of DMDD could persist into adulthood [6]. Copeland et al. [6] showed that as adults, youths with DMDD present a higher level of adverse health outcomes, financial problems, police contact, and lower educational attainment than those with any other childhood-onset psychiatric disorders.

Despite all of these findings, the DMDD diagnosis remained a controversial diagnosis [12]. Most youths with DMDD meet the criteria for another psychiatric disorder, especially an oppositional defiant disorder (ODD). As irritability, the core symptom of DMDD is a criterion for almost 12 psychiatric disorders in the DSM-5, a significant overlap exists between DMDD and other psychiatric disorders. The authors then questioned the validity of DMDD as a unique and independent diagnosis [13]. While the proponents stressed the specific course of irritability symptoms in DMDD (i.e., age at the onset before 10, chronic course) and the risk of developing depressive disorders in adulthood, the opponents have pointed out the lack of empirical evidence and the risk of hidden potentially treatable associated conditions (e.g., providing a cognitive behavioral therapy for anxiety symptoms or a psychostimulant for attention deficit disorder with hyperactivity, ADHD) [14].

A systematic review and meta-analysis were conducted to examine heterogeneous findings about the epidemiology of DMDD. Questions about the comorbidity of youths with DMDD were raised as one of the main concerns about the diagnosis validity. To address this issue, a meta-analysis was regarded as an adequate methodological strategy to help overcome the limitations reported in previous studies, especially the small sample sizes, the variability in the study setting, and the DMDD conceptualization. The research was planned to answer the following questions:

- What is the pooled prevalence of DMDD in community-based samples? What is the pooled prevalence of DMDD in clinical samples? What socio-demographic factors moderated the prevalence of DMDD? How does the adherence to DSM-5 criteria influence the prevalence rate?
- What are the rates of co-occurring psychiatric or neurodevelopmental disorders with DMDD? Do they differ across contexts (i.e., in the general population, in help-seeking samples referred to outpatient or inpatient facilities)?

The variability observed in the reviewed studies will be critically discussed in light of longitudinal research findings on chronic irritability in the general population or at-risk samples.

## Methods

Preferred reporting items for systematic reviews and meta-analyses (PRISMA) 2020 guidelines are followed in this report [15]. The protocol was registered online with the International Prospective Register of Systematic Reviews (PROSPERO Registration number: CRD42023427721) and can be accessed at https://www.crd.york.ac.uk/PROSPERO/display_record.php?RecordID=427721.

### Search strategy

**Figure 1.**
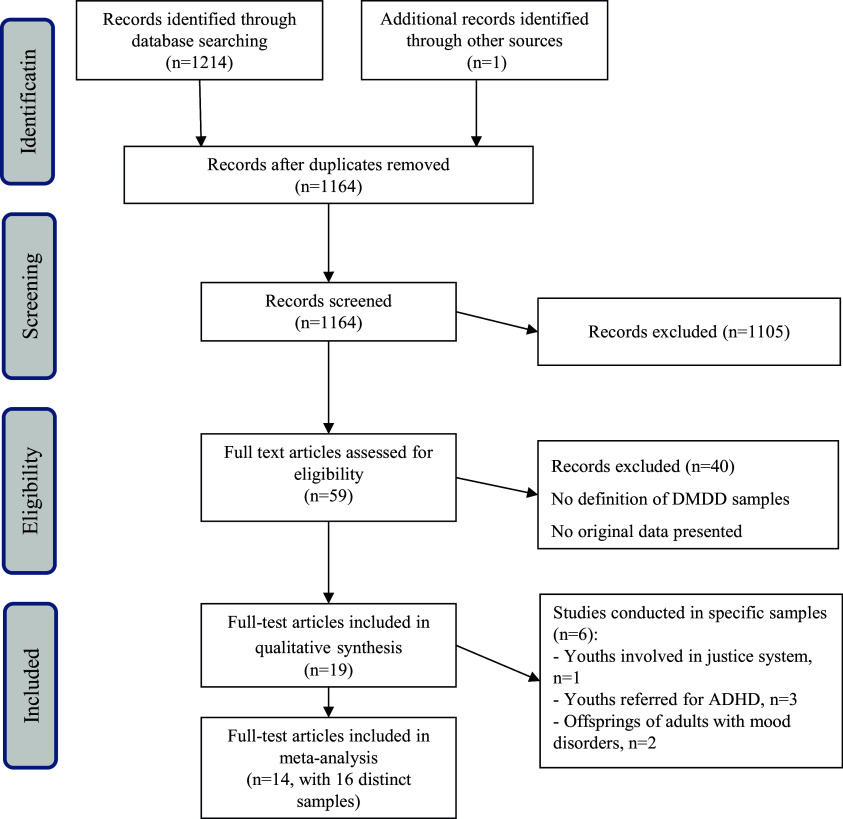
PRISMA flow-chart.

The PubMed and PsychINFO electronic bibliographic databases were searched from May 2013 (i.e., the publication of the DSM-5) to July 2023, and data were first extracted in September 2023. An updated database search was conducted in November 2024. The search strategy included the terms shown in Table 1, which were combined using database-specific filters when these were available. The flow chart shown in Figure 1 complies with PRISMA recommendations. The references of the selected articles were also hand-searched, and prior recent reviews’ reference lists were also reviewed, such as [12, 16, 17].

**Table 1.** General strategy for the review search terms

Domain	Words
Age group	“children” OR “adolescents” OR “teen*” OR “youths”
Disorders	“disruptive mood dysregulation disorder” OR “irritability”
Other	“assessment” OR “diagnosis” OR “measure*” OR “questionnaire” OR “psychometr*” OR “interview” OR “screen” OR “scale” OR “checklist” OR “valid*” OR “prevalence” OR “incidence” OR “comorbidity” OR “epidemiology”
Exclusion filter	limited to English language; May 2013–November 2024; age 0 to 18 years

*Note:* Some of these terms were slightly differed according to the electronic bibliographic database

### Selection criteria

One author screened the titles and abstracts of articles. Ambiguous papers were a priori included. Two authors reviewed all selected full-text articles for eligibility. The agreement between the two raters for the final selection based on full-text articles assessed for eligibility was 89.74%, *k* = 0.69.

All studies where information was available about the prevalence or comorbidity rates of DMDD were included, whatever the authors’ main aims. Other clinical entities that had previously been used to catch youths with severely impairing and persisting dysregulated mood were not included (i.e., *Severe Mood Dysregulation, Temper Dysregulation Disorder with Dysphoria, Bipolar Disorder Not Otherwise Specified*, the large phenotype of pediatric bipolar disorder coined by the National Institute for Health and Care Excellence in England, the *Child Behavior Checklist – Juvenile Bipolar Disorder Profile*, further relabeled *CBCL-Dysregulation Profile*). We decided not to include such a large spectrum of irritability-related clinical entities because the aim was to investigate the epidemiology of DMDD as defined per the DSM-5.

The following studies were excluded:

1. studies conducted in adults
2. studies where data from pediatric (<18 years old) and adult samples were pooled
3. studies with no original data (e.g., abstract, editorial). When several studies were published on the same cohort, the largest study was considered (e.g., information about DMDD prevalence from the 2004 Pelotas Birth Cohort Study was reported in [18–20]). Systematic reviews and meta-analyses were examined for references but not included.

Studies conducted on special populations (e.g., offspring’s of adults with mood disorders) were included for qualitative but not quantitative analyses. Regarding the scope of our review on prevalence and comorbidity rates, this category was regarded as too heterogeneous to enable pooled analyses.

### Data extraction method

For each selected study, the following information was noted using a previously tested data extraction form: (i) participants’ features (sample size, gender, mean age, ethnic status, treatment settings, and location); (ii) diagnostic assessment and retained criteria for DMDD; (iii) prevalence estimates including the timeframe of prevalence estimate (e.g., point prevalence, annual prevalence), any prevalence estimates reported stratified by age, sex, or location; and (iv) comorbidity rates of associated psychiatric and neurodevelopmental disorders (primary psychiatric diagnoses, measurement tools). The comorbidity rates with ODD and bipolar disorders have not been assessed as they both constitute exclusion criteria for DMDD in the DSM-5.

Once identified, the methodological quality of each article was examined using the quality assessment instrument for prevalence studies published by Boyle [21], such as presented in Labelle, Pouliot [22] (Table 2). Studies were assigned one point for each positive following item: (a) definition of the target population; (b) probability sampling or entire population surveyed; (c) response rate above 80%; (d) description of non-responders; (e) the sample was representative of the target population; (f) standardized data collection; (g) strict adherence to DMDD criteria (1: if all DSM-5 criteria/0: other cases); and (h) the prevalence estimates provided with confidence intervals and detailed by subgroups. Two authors separately coded each study across the eight domains of bias. In case of discrepancies, the two reviewers chose the final score after discussion. Inter-rater reliability was substantial *ICC* = 0.73 (95% CI, 0.34-0.91) among the raters.

**Table 2.** Risk of bias in reviewed studies considered for quantitative analysis

Authors	Definition of the target population	Probability sampling or the entire population surveyed	Response rate above 80%	Description of non-responders	A sample representative of the target population	Standardized data collection	Strict adherence to diagnosis criteria	Confidence intervals and subgroups analysis	Overall score
Margulies et al. [29]	1	0	0	0	1	1	0	1	4
Axelson et al. [5]	0	0	0	1	1	1	0	0	3
Copeland et al. [8]	1	0	1	1	1	1	0	1	6
Dougherty et al. [9]	0	1	0	1	1	1	0	1	5
Mayes et al. [26]	1	0	0	1	1	1	0	0	4
Althoff et al. [10]	1	0	1	1	1	1	1	1	7
Tufan et al. [68]	0	0	1	0	0	0	1	0	2
Freeman et al. [33]	1	1	0	0	1	1	0	1	5
Tüğen et al. [31]	1	0	1	0	1	1	0	0	4
Chen et al. [30]	1	0	0	0	1	1	1	1	5
Benarous et al. [57]	0	1	1	1	0	1	1	1	6
Benarous et al. [69]	0	1	1	1	0	1	1	1	6
Bauer et al. [20]	1	1	1	1	1	1	1	1	10
Coldevin et al. [70]	1	1	0	0	0	1	1	1	5

*Note.* We reviewed 14 different articles, for 16 distinct samples.

### Meta-analysis

We gathered the studies based on the population studied (community-based versus clinical samples) during the data extraction. Prevalence figures and 95% confidence intervals (CIs) were extracted or calculated from the available data using Wilson’s method, which is regarded as having better coverage rates for small samples [23].

Heterogeneity between estimates was assessed using the *I* ^2^ statistic and a homogeneity test from a *χ* ^2^ statistic. For the *I* ^2^ statistic, a value above 75% indicates high heterogeneity. Considering putative within-study variability, a random effect model was used. Potential influences on prevalence estimates were investigated using subgroup analyses and meta-regression. The influence of the variables identified a priori as possible sources of variation in the estimates of prevalence were examined: (1) the strictness of adherence to DSM diagnosis criteria with three categories ([all DSM criteria] versus [all DSM criteria except exclusion criteria for psychiatric comorbidity] versus [all DSM criteria except exclusion criteria for psychiatric comorbidity and age criteria (i.e., age at the onset before 10 and at least 6 year old)]), (2) geographical area (US versus other countries), (3) data collection method ([self-completed questionnaire] versus [data collection method that required some form of human interaction such as a semi-structured interview or clinician questionnaire]), (4) mean age of participants, (5) gender ratio of participants, (6) ethnic status (proportion of white), and (7) the overall score for the risk of bias.

**Figure 2.**
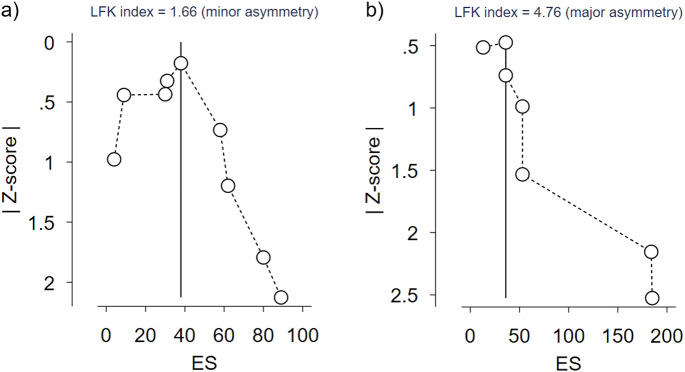
Doi plot of studies measuring the prevalence of DMDD in (a) community-based samples and (b) clinical samples.

Considering the limitation of funnel plots to estimate publication bias in a meta-analysis of proportions [24], doi plots and the LFK index were performed in the community-based samples (Figure 2) and clinical samples (Figure 2b). A Doi plot shows normal-quantile against effect size. It is inspected visually by looking at the dots representing individual studies and their arrangement. As for the funnel plot, an asymmetry of the figure suggests publication bias. The LFK index is a quantitative interpretation of the Doi plot; a value outside the range of −1 to +1 is considered significant. Analyses were computed using the software Stata-16 [25].

## Results

The systematic review yielded 1,214 hits, and 1,105 hits were excluded based on the information in the title or abstract. The full texts of the remaining 59 hits were critically reviewed, excluding another 40 articles. Of the final 19 reviewed studies, 14 studies presented data directly exploitable for pooled analysis based on 16 distinct samples.

### Description of the studies

Data on the epidemiology of DMDD was assessed in nine distinct community-based samples. Of note, the article published by Copeland et al. [8] presented data from three distinct cohorts. Seven studies presented data on the epidemiology of DMDD in clinical samples (Table 3).

**Table 3.** Reviewed studies in community-based samples and clinical sample

Authors/years /samples studied	Demographic features	Diagnostic assessment	DSM-5 criteria
Community-based samples			
Copeland et al. [8] Great Smoky Mountain Study (2013)	US N = 1,420 *M* age = 13.7 (2.0) [9–17] F = 49.2% White = 89.8%	Retrospective diagnosis Items from a PSCI CAPA	A-B: items from the ODD section “*temper tantrums”* and *“outbursts* ‘ C (frequency criteria): yes D items from the depression section “*depressed, sad, irritable, or angry mood*” or “*low frustration threshold*” E (duration criteria): yes F (cross-domain impairment): yes G (age at diagnosis): yes, by default H (age at onset): yes I (exclusion criteria manic symptoms): yes J -K (other exclusion criteria): yes
Copeland et al. [8] The Duke Preschool Anxiety Study	US N = 918 *M* age = 3.9 (1.3) [2–6] F = 51.8% White = 62.1%	Retrospective diagnosis Items from a PSCI CAPA	A-B: items from the ODD section “*temper tantrums”* and *“outbursts* ‘ C (frequency criteria): yes D items from the depression section “*depressed, sad, irritable, or angry mood*” or “*low frustration threshold*” E (duration criteria): yes F (cross-domain impairment): yes G (age at diagnosis): no H (age at onset): yes I (exclusion criteria manic symptoms): yes J -K (other exclusion criteria): yes
Copeland et al. [8] The Caring for Children in the Community study	US N = 920 *M* age = 14.2 (3.4) [9–17] F = 50.0% White = 41.0%	PAPA DSM-IV criteria	A-B: items from the ODD section “*temper tantrums”* and *“outbursts* ‘ C (frequency criteria): yes D items from the depression section “*depressed, sad, irritable, or angry mood*” or “*low frustration threshold*” E (duration criteria): yes F (cross-domain impairment): yes G (age at diagnosis): yes, by default H (age at onset): yes I (exclusion criteria manic symptoms): yes J -K (other exclusion criteria): yes
Dougherty et al. [9] Stony Brook Temperament Study	US N = 462 Age *M* = 6.1 (0.4) F = 45.9% No ethnic data	Retrospective diagnosis Items from a PSCI PAPA DSM-IV criteria	A-B: items from the ODD section “*temper tantrums and outbursts* ‘ D: items from depression ‘*anger, irritability, annoyance*, or “*low frustration tolerance*” ≥*45 times in the past 3 months* C (frequency criteria): yes E (duration criteria): yes F (cross-domain impairment): yes G (age at diagnosis): yes, by default H (age at onset): yes I (exclusion criteria manic symptoms): no J -K (other exclusion criteria): no, “in order to examine overlap with other psychiatric disorders”
Mayes et al. [26] School-based sample	US N = 665 Age *M* = 8.7 (1.7) [6–12] F = 47.4% White = 80.5%	Questionnaires were sent home to the parents of every elementary school Subjective maternal rating of two major symptoms of DMDD PBS	A-B-D: “*irritable, gets angry or annoyed easily*’” and “*loses temper, has temper tantrums*” as *often* or *very often* a problem C (frequency criteria): no (“*often”* or “*very often*”) E (duration criteria): no, 2 months F (cross-domain impairment): no G (age at diagnosis): no H (age at onset): no I (exclusion criteria manic symptoms): no J -K (other exclusion criteria): no
Althoff et al. [10] National Comorbidity Survey-Adolescent Supplement Cross-sectional	US N = 6,483 Age *M* = 15.11 (X) [13–18] F = 51.4% White = 65.6%	Retrospective diagnosis CIDI-III PSAQ DSM-IV criteria	A-B-D: “*lose temper, tantrums, angry outburst, anger attack*” and “*fight with others or bullies them*” (not really irritability) C (frequency criteria): +/− (156 per year of physical or verbal threats) E (duration criteria): no F (cross-domain impairment): yes (if one of the 6 items is true, but it should involve more than one domain) G (age at diagnosis): by default, as the participants are 13–18 years old H (age at onset): yes I (exclusion criteria manic symptoms): +/−, manic symptoms but not duration criteria J -K (other exclusion criteria): no, “in order to examine overlap with other psychiatric disorders”
Tüğen et al. [27] School-based sample	Turkey N = 453 Age *M* not specified No F data No ethnic data	CBCL DSM–5 criteria	A-B-D: yes C (frequency criteria): yes E (duration criteria): yes F (cross-domain impairment): yes G (age at diagnosis): yes H (age at onset): yes I (exclusion criteria manic symptoms): yes J -K (other exclusion criteria): yes
Chen, Chen et al. (2019) [28] School-based national	Taiwan N = 4,816 Age *M* not specified F = 48% No ethnic data	K-SADS-PL	A-B-D: yes C (frequency criteria): yes E (duration criteria): yes F (cross-domain impairment): yes G (age at diagnosis): yes H (age at onset): yes I (exclusion criteria manic symptoms): yes J -K (other exclusion criteria): yes
Bauer et al. [20] 2004 Pelotas Birth Cohort	Brazil N = 3,367 At age 11 F = 48.1% White = 62%	DAWBA Clinical interview, for DSM-IV, DSM–5, and ICD–10 psychiatric diagnoses for children aged 5–17 years	A-B-D: yes C (frequency criteria): yes E (duration criteria): yes F (cross-domain impairment): yes G (age at diagnosis): yes H (age at onset): yes I (exclusion criteria manic symptoms): yes J -K (other exclusion criteria): yes
CLINICAL SAMPLES			
Margulies et al. [29]	US N = 82 Age *M* = 9.8 (2.1) [5–12] F 33.2% White 75.6% Inpatient psychiatric unit (a 10-bed university hospital children’s) One site	CASI CMRS-P Adhoc inventory of rage behaviors DSM-IV criteria	A-B-D: items from the ODD and mania section: “*irritability”* and “*explosiveness”* as *often* or *very often* AND *observed irritability and explosiveness* by the medical and unit director C (frequency criteria): no E (duration criteria): yes F (at least two settings): no G (age at diagnosis): yes, by default H (age at onset): yes I (exclusion criteria manic symptoms): yes J -K (other exclusion criteria): yes
Axelson et al. [5]	US N = 706 Age *M* = 9.4 (1.9) [6–12] at baseline] F = 32.4% White = 64.4% Psychiatric outpatient population Longitudinal Assessment of Manic Symptoms (24 months follow-up) 9 centers	Retrospective diagnosis K-SADS-PL YMRS CMRS-P DSM-IV criteria	A-B: items from the depression, ODD, or mania section: “*loses temper*” and “*severe temper outbursts*” 2–5 times per week C (frequency criteria): yes D: “*easily annoyed or angered*” and “*angry or resentful*” as daily or almost daily E (duration criteria): no, 6 months F (cross-domain impairment): yes G (age at diagnosis): yes, by default H (age at onset): no I (exclusion criteria manic symptoms): no “whether the DMDD phenotype can be delimited from BD is a question to be evaluated” J -K (other exclusion criteria): yes, except ODD to measure comorbidity
Freeman et al. [30]	US N = 597 Age *M* = 10.6 (3.4) [5–18] F 39% White 6% Outpatient community center One site	Retrospective diagnosis Items from KSADS-PL CBCL YSR TRF YMRS CDRS DSM-IV criteria	A-B: items from the depression and mania section: “*loses temper*” and “*severe temper outbursts*” 2–5 times per week C (frequency criteria): yes D: “*easily annoyed or angered*” and “*angry or resentful*” as daily or almost daily E (duration criteria): no, 6 months F (cross-domain impairment): yes G (age at diagnosis): yes, by default H (age at onset): no I (exclusion criteria manic symptoms): yes, “elated mood” symptom rated as “mild” or greater J -K (other exclusion criteria): yes
Tufan et al. [31]	Turkey N = 403 Age *M* = 9.0 (2.5) [6–17] F 22.2% No ethnic data Inpatient psychiatric unit Two sites	Retrospective diagnosis CS Parental-report symptom	A-B-D: “*ready to pick up a fight, quick to anger”* as “much” or “very much,” “*is cranky and sullen*” as “much” or “very much” C (frequency criteria): yes, based on chart review E (duration criteria): yes, based on chart review F (cross-domain impairment): no G (age at diagnosis): yes, by default H (age at onset): no I (exclusion criteria manic symptoms): no J -K (other exclusion criteria): no
Benarous et al. [32]	Canada N = 165 Age *M* = 13.7 (0.3) [5–21] F 59.4% No ethnic data Outpatient community center and specialized mood clinics Two sites	Retrospective diagnosis KSADS Observed by medical staff	A-B-D: clinical grid analysis C (frequency criteria): yes, but assessment E (duration criteria): yes F (cross-domain impairment): yes G (age at diagnosis): yes H (age at onset): yes I (exclusion criteria manic symptoms): yes J -K (other exclusion criteria): yes
Benarous et al. [11]	Paris N = 191 Age *M* = 14.71 ± 1.71 [12–18] F 41% No ethnic data Inpatient One site	Retrospective diagnosis KSADS Observed by medical staff	A-B-D: clinical grid analysis C (frequency criteria): yes, but assessment E (duration criteria): yes F (cross-domain impairment): yes G (age at diagnosis): yes H (age at onset): yes I (exclusion criteria manic symptoms): yes J -K (other exclusion criteria): yes
Coldevin et al. [33]	Norway N = 218 Age *M* = 9.6 ± 1.8 [6–12.9] F 40% No ethnic data Outpatient Three sites	KSADS	A-B-D: yes C (frequency criteria): yes E (duration criteria): yes F (cross-domain impairment): yes G (age at diagnosis): yes H (age at onset): yes I (exclusion criteria manic symptoms): yes J -K (other exclusion criteria): yes

Abbreviation: CAPA, Child and Adolescent Psychiatric Assessment; CASI, Child and Adolescent Symptom Inventory; CBCL, Child Behavior Checklist; CDRS, Child Depression Rating Scale; CIDI-III, Composite International Diagnostic Interview, version 3; CMRS-P, Child Mania Rating Scale Parent version; CS, Conners Scale; DAWBA, Development and Well-Being Assessment; K-SADS-PL, The Schedule for Affective Disorders and Schizophrenia for School-Age Children-Present and Lifetime version*;* PAPA, Preschool Age Psychiatric Assessment; PBS, Pediatric Behavior Scale; PSAQ, Parental Self-Administrated Questionnaire; PSCI, parent-reported structured clinical interview; *TRF, Teacher’s Report Form a parallel form of the CBCL fulfilled by teachers;* V-DISC, Voice Diagnostic Interview Schedule for Children; YMRS, Young Mania Rating Scale; YSR, Youth Self-Report *a parallel form of the CBCL fulfilled by the youth.*

Five studies were conducted in at-risk samples, more precisely among justice-involved youths [34], youths referred for ADHD [35, 36], and offspring’s of adults with mood disorders [37, 38].

### Prevalence

#### Community-based samples

The pooled prevalence of DMDD in community-based samples was 3.33% (95% CI, 1.43–5.96). There was an apparent heterogeneity across included studies, suggesting the use of a random-effect meta-analysis model (*I* ^2^ = 98.57%, *χ* ^2^ (8) = 558.93, *p* < .001).

**Figure 3.**
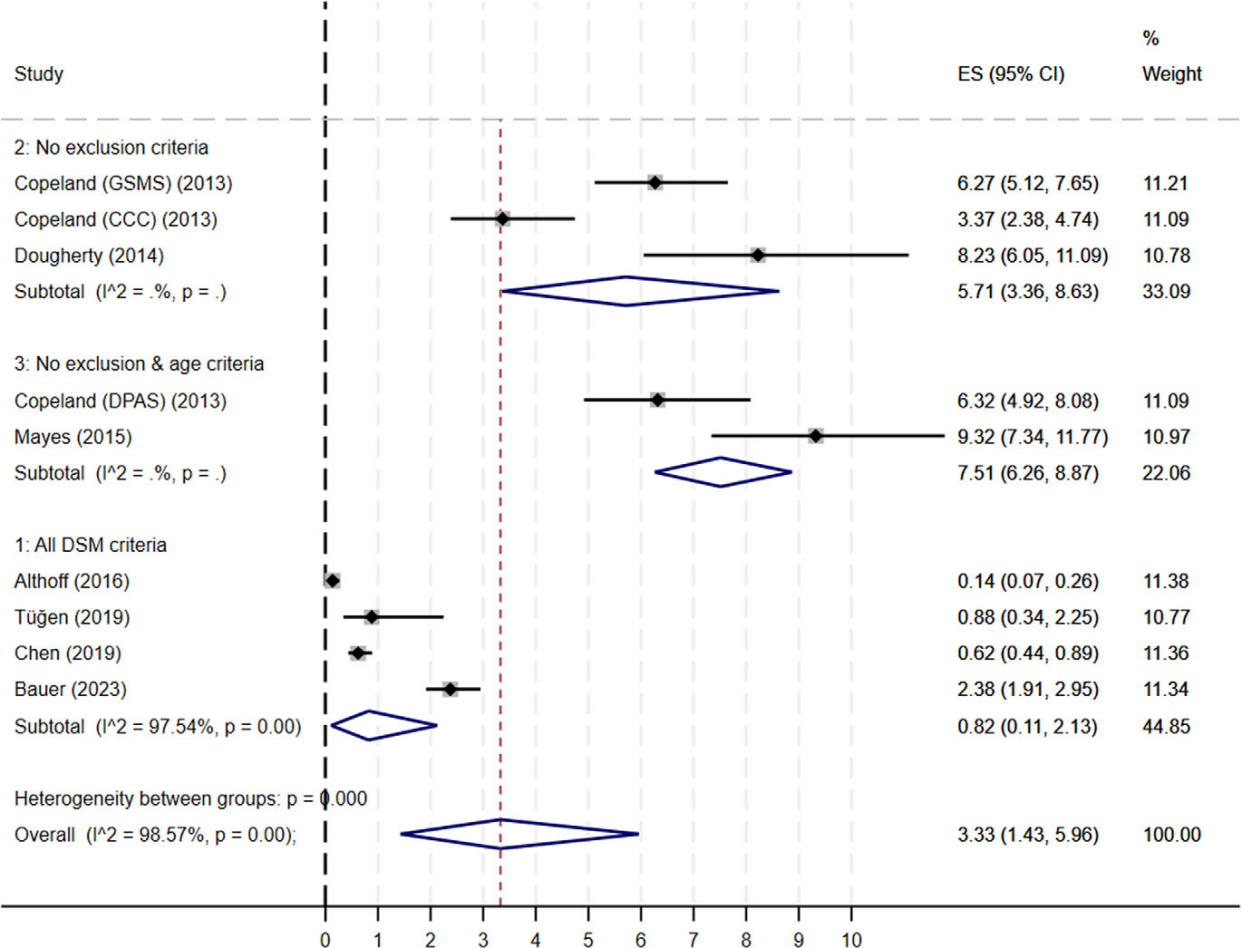
Forest plot of studies measuring the prevalence of DMDD in community-based samples: subgroup analysis based on the number of DSM criteria used. *Note.* The number (1 to 3) refers to the different ways the DMDD was identified in the reviewed studies (1 = studies using all DSM criteria, 2 = studies using all DSM criteria except exclusion criteria for psychiatric comorbidity (i.e., bipolar disorder), 3 = studies using all DSM criteria except exclusion criteria for comorbidity and age criteria (age at the onset before 10 and at least 6-year-old).

*Subgroup analyses*: The difference in the strictness of adherence to the DSM diagnosis criteria was associated with statistically significant heterogeneity (Figure 3). The pooled prevalence of DMDD was 0.82% (95% CI, 0.11–2.13) in studies where strict adherence to all DSM-5 criteria was used. The pooled prevalence in studies using all DSM criteria except exclusion criteria for psychiatric comorbidity was 5.71% (95% CI, 3.36–8.63). The pooled prevalence in studies using all DSM criteria except exclusion criteria for psychiatric comorbidity and age criteria was 7.51% (95% CI, 6.26–8.87). The study location did not significantly influence the prevalence.

*Meta-regressions:* Meta-regression analysis showed that the mean prevalence of DMDD was substantially influenced by the age of participants (i.e., lower age had higher prevalence) but not by other participants’ sociodemographic features such as gender ratio, ethnic status, and the overall quality of the study (Table 4).

**Table 4.** Summary effect sizes, measure of heterogeneity, moderators, and bias for the prevalences

	Community-based samples	Clinical samples
Number of studies	9	7
Number of participants	19,504	2,362
Random pooled ES [95% CI]	3.33 [1.43, 5.96]	21.88 [15.47, 29.05]
Heterogeneity: *I* ^2^	98.57%	93.30%
Moderation effects		
Age	*β* = −0.01, *p* = .049	*β* = 0.01, *p* = .434
Gender ratio	*β* = −0.56, *p* = .419	*β* = 0.01, *p* = .218
Ethnic status (white proportion)	*β = −0.01, p = *.076**	*β* = −0.01, *p* = .857
Risk of bias	*β* = −0.01, *p* = .076	*β* = 0.03, *p* = .163
Subgroup analysis		
Adherence to DSM	*z* (2) = 36.81, *p* < .001	*z* (2) =3.42, *p* = .180
Study location (US versus non-US)	*z* (1) =2.97, *p* = .080	*z* (1) =0.82, *p* = .360
Setting	–	*z* (1) =1.70, *p* = .190
LFK index	1.66 (minor asymmetry)	4.76 (major asymmetry)

#### Clinical samples

**Figure 4.**
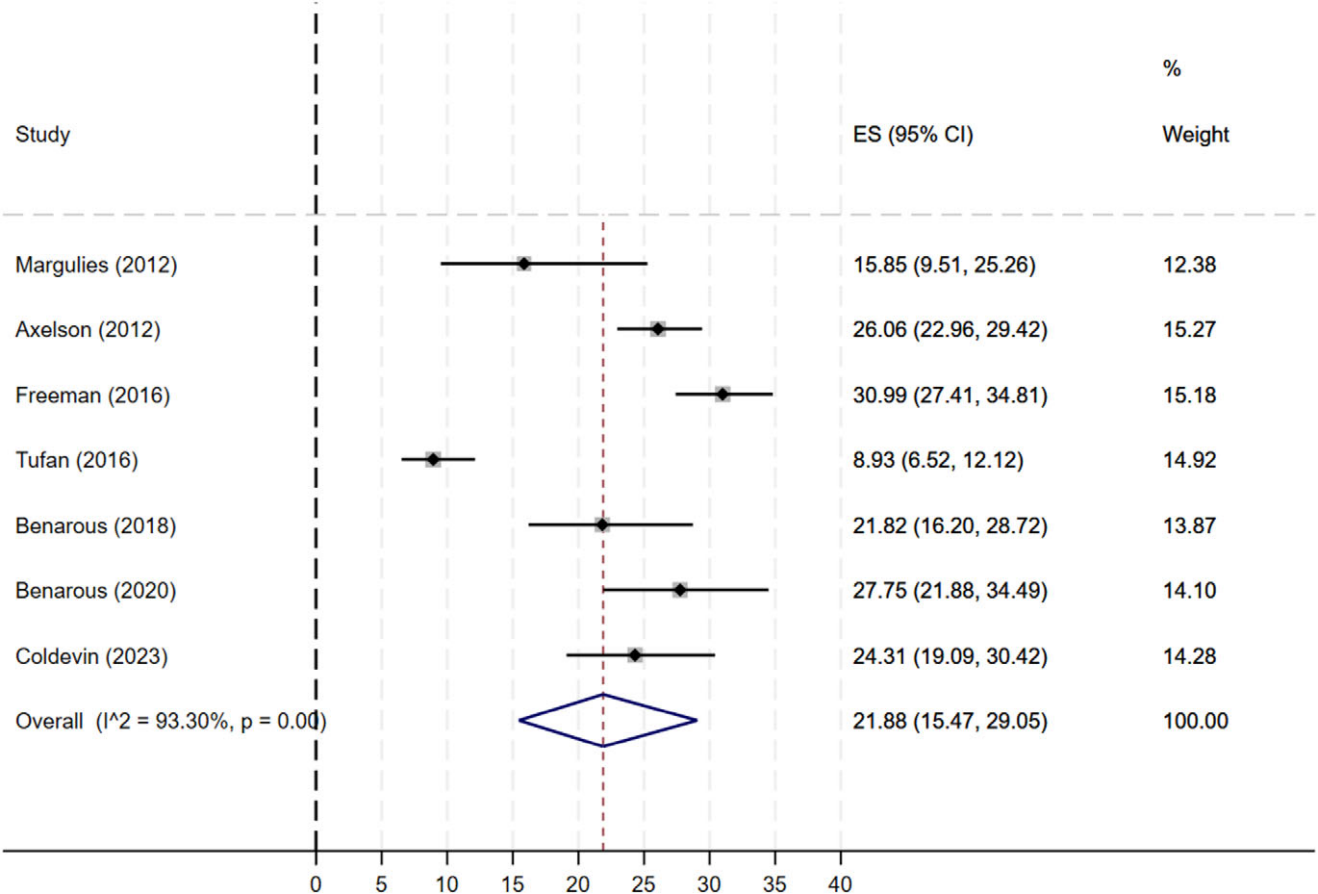
Forest plot of studies measuring the prevalence of DMDD in clinical samples.

The pooled prevalence of DMDD in clinical samples was 21.88% (95% CI, 15.47–29.05). There was an apparent heterogeneity across included studies, suggesting the use of a random-effect meta-analysis model (*I* ^2^ = 93.30%, *χ* ^2^ (6) = 89.62, *p* < .001). Visual inspection of the forest plot (Figure 4) showed that the confidence intervals of the prevalence reported by Tufan (2016) did not overlap with others’ reported prevalence.

*Subgroup analyses:* The prevalence of DMDD in clinical samples was not substantially influenced by the strictness of adherence to the DSM diagnosis criteria, the setting of the study (inpatient versus outpatient), and the study location (Table 4).

*Meta-regressions:* Meta-regression analysis showed that the mean prevalence of DMDD in clinical samples was not substantially influenced by participants’ sociodemographic features, such as the age of participants, gender ratio, ethnic status, and the overall quality of the study (Table 4).

### Comorbidity rates

#### Anxiety disorders

The prevalence of anxiety disorders in youths with DMDD in community-based samples was 28.41% (95% CI, 7.32–55.66, *k* = 6, *I^2^* = 94.36%, *χ* ^2^ (5) = 88.59, *p* < .001). The prevalence of anxiety disorders in youths with DMDD in clinical samples was 27.68% (95% CI, 15.67–41.49, *k* = 6, *I^2^* = 88.87%, *χ* ^2^ (5) = 44.92, *p* < .001).

#### Depressive disorders

The prevalence of depressive disorders in youths with DMDD in community-based samples was 23.79% (95% CI, 13.67–35.50, *k* = 6, *I^2^* = 72.03%, *χ* ^2^ (5) = 17.88, *p* < .001). The prevalence of depressive disorders in youths with DMDD in clinical samples was 20.37% (95% CI, 11.11–31.41, *k* = 6, *I^2^* = 83.92%, *χ* ^2^ (5) = 31.10, *p* < .001).

#### Conduct disorders

The prevalence of conduct disorder in youths with DMDD in community-based samples was 22.37% (95% CI, 16.42–28.91, *k* = 3, *I^2^* = 0%, *χ* ^2^ (2) = 0.18, *p =* .920). The prevalence of conduct disorders in youths with DMDD in clinical samples was 12.94% (95% CI, 6.03–21.70, *k* = 5, *I^2^* = 78.36%, *χ* ^2^ (4) = 18.49, *p* < .001). It was not assessed in community-based samples.

#### ADHD

The prevalence of ADHD in youths with DMDD in community-based samples was 13.47% (95% CI 5.48–23.84, *k* = 6, *I^2^* = 73.45%, *χ* ^2^ (5) = 18.83, *p <* .001). The prevalence of ADHD in youths with DMDD in clinical samples was 61.12% (95% CI, 45.27–75.91, *k* = 7, *I^2^* = 91.60%, *χ* ^2^ (6) = 71.44, *p* < .001).

#### Trauma and stressors-related disorders

The prevalence of trauma and stressors-related disorders in youths with DMDD in clinical samples was 29.19% (95% CI, 20.05–39.22, *k* = 2, z = 9.49, *p <* .001).

### Narrative review of studies on at-risk samples

In a study conducted on 2,498 youths involved in the US justice system (mean age 15.8, 77% boys) Mroczkowski et al. [34] reported a prevalence of DMDD at 3.3% based on a retrospective diagnosis using the ODD section of the Voice Diagnostic Interview Schedule for Children (V-DISC) to measure irritability symptoms.

Mulraney et al. [36] examined the comorbidity and correlates of DMDD in 6–8-year-old children with ADHD recruited in several Melbourne (Australia) schools screened with the Conners 3 ADHD index and diagnosed with the DISC-IV. Twenty-two percent of recruited children (*n* = 39/179) had proxy criteria for DMDD, with an extensive majority also meeting criteria for ODD (90%) and for 41% of them anxiety disorders. Özyurt et al. [35] compared 22 children with both DMDD and ADHD to 30 with only ADHD and 60 healthy controls. The authors reported more social cognition difficulties in the group with both conditions based on a questionnaire (i.e., the KaSi Empathy Scale) and a neuropsychological task (i.e., the Reading Mind in the Eyes Test).

In a sample of 12–16-year-old adolescent offsprings of adults with mood disorders (*n* = 62), Topal et al. [38] reported five cases of lifetime DMDD using the K-SADS-PL semi-structured interview. In contrast, Perich et al. [37] found no subject fulfilling current or lifetime DMDD criteria in an Australian sample of 29 offspring of adults with bipolar disorders.

## Discussion

### Main findings

#### Prevalence of DMDD

The evidence reviewed strongly suggests that DMDD is prevalent, concerning 3.3% of children and adolescents in community-based samples. Increasing prevalence moving from community-based to clinical settings was marked, with a prevalence of DMDD in clinical samples estimated at 21.9%. The first reason for this over-presentation of DMDD in clinical samples is that irritability-related behaviors (e.g., aggressive, reactive, hostile behaviors, self-aggressive behavior) are frequent reasons parents seek care for their children [39, 40]. As irritability is “*at the crossroads of internalized and externalized disorders*” [41], the high prevalence of DMDD in clinical settings could reflect a Berkson bias since both difficulties can lead to referral [42]. Of note, the pooled prevalence of DMDD in community-based samples reported here was higher than the range of prevalence of major depressive disorder in children and adolescents based on large national representative samples (0.14%–2.2%) [43–45].

Substantial heterogeneities between studies were found both in community-based and clinical samples. An important source of variability was how much the studies adhered to the diagnostic criteria for DMDD, as only a minority used a definition of DMDD that meets all criteria (4/9 for community-based samples, 3/7 for clinical samples). For example, the DSM-5 states that “*[DMDD’s] symptoms are not occurring exclusively during a psychotic or mood disorder or are better accounted for by another disorder.*” The cross-sectional nature of the data collected in the reviewed studies and the proxy measures frequently used for DMDD make it highly complex to determine on which extent the co-occurring rates reported are artifactual or reflect true comorbidities.

The prevalence of DMDD also widely varies based on adherence to time-related diagnosis criteria, i.e., symptoms duration, age at diagnosis, and age at symptom onset. Several longitudinal studies showed that the level of irritability in the general population tends to peak between 2 and 6 years of age before decreasing for most children in the general population after age [46–51]. These findings could explain the significant relation reported between the age of the participants and the prevalence of DMDD in the community-based samples reviewed in our study. Based on this, the inclusion of the studies by Dougherty et al. [9] and the cohort *Caring for Children in the Community* in Copeland et al. [6] can be questioned as participants were preschoolers while subjects have to be aged at least 6 years to make a diagnosis of DMDD [1]. Finally, in the DSM-5, the onset of temper outbursts should occur before the age of 10 years. An issue worth considering to help clinicians distinguish between DMDD and episodic mood disorders. The only study conducted in a community-based sample that did not retain the age at symptom onset criteria [26] reported a much higher prevalence of DMDD compared to other studies (Figure 1).

The meta-regression analyses conducted on data from clinical samples did not find any significant effect of the participants’ sociodemographic characteristics on the prevalence of DMDD. Unlike our expectations, no frequency gradient was found from outpatient to inpatient facilities. The chronic course of DMDD symptoms (and then the lack of sudden change in functioning) may discourage clinicians from referring this patient to full-time hospitalization, which is usually orientated towards crisis interventions in most developed countries [52].

#### Comorbid psychiatric disorders

The association between DMDD and anxiety and depressive disorders was consistent with cumulative evidence supporting that DMDD predicts the risk for emotional disorders [53]. Using data from the *Longitudinal Assessment of Manic Symptoms* study to examine the 2-year outcome of subjects with DMDD Axelson, Findling [5] found a higher risk of depressive disorder (*OR* = 1.29) and anxiety disorder (*OR* = 1.45). In the study by Copeland et al. [6] conducted on the 1,420 participants of the *Great Smoky Mountain Study* followed for 25 years, the occurrence of depressive disorder was 4.6 times more frequent in adulthood among young people with DMDD, and anxiety disorders 3.2 times more frequent. The link between DMDD and depressive disorders has also been documented in terms of family studies, genetic linkage analysis, and neurocognitive abnormalities [41]. In our meta-analysis, between 20% and 24% of young people with DMDD have an associated depressive disorder, and 27%–29% have an associated anxiety disorder. The association with conduct disorders is estimated between 12% and 23%. This figure is lower than those reported in previous studies where conduct disorders and ODD are usually combined and investigated under the category “disruptive behavioral disorder” (the association with intermittent explosive disorders was never examined).

The association between DMDD and ADHD described in previous reports [54, 55] varies widely between studies, with an average of 13% in the community-based samples and 62% in the clinical samples. Although irritability is not a diagnostic criterion for ADHD, temper tantrums and emotion regulation difficulties are frequently reported in ADHD patients [54]. Comparable cognitive impairments were also reported for both disorders, in particular in executive function [7]. A high level of comorbidity between the two disorders led some authors to view DMDD as a subtype of ADHD [56]. As nearly 87% of young people in the community-based samples with DMDD do not have ADHD, this hypothesis can reasonably be ruled out based on our review. Of note, the gap in the comorbidity rates observed in community-based and clinical populations is more marked for ADHD than for other disorders. One may hypothesize that patients with both disorders are at particular risk of suicidal behaviors requiring admission to an inpatient facility due to the synergic effect of emotional lability and impulsivity [40, 57]. As participants in clinical samples were mostly included in university teaching hospitals and were usually experts in neurodevelopmental disorders, this finding may also partly reflect a selection bias.

Nearly 29% of youths with DMDD in clinical samples had stress and trauma-related disorders. This result remains to be confirmed as it is supported by only two studies conducted by the same research team. In this vein, Wang et al. [58] stressed the need to gain more information about the relationship between DMDD and traumatic experiences in community-based samples. In the authors’ response, Bauer et al. [20] presented additional analyses from the Brazilian Pelotas 2004 birth cohort (*N* = 4,229). Exposure to trauma up to the age of 11 years was associated with a 1.70 times higher risk of developing DMDD after adjustment to pre-existing psychiatric symptoms and other potential confounding factors. Some studies conducted in samples at high risk of being exposed to adverse childhood experiences found a high frequency of DMDD, such as young people involved in judicial structures [34] or child protection services [59].

### Limitations

Some limitations of this review warrant discussion. Firstly, a substantial amount of the heterogeneity among the studies remained unexplained by the variables examined. The random-effects meta-regressions analyses conducted may have low power, particularly in the presence of large unexplained heterogeneity [60]. Potentially underpowered sub-group analyses and meta-regressions should make us cautious about interpreting these specific analyses. The Cochrane Handbook for Systematic Reviews of Interventions recommends a minimum of 10 studies to compute meta-regression or subgroup analysis, slightly above the number of studies here. However, the assumption that adherence to DSM criteria, especially age, is an important factor in understanding the heterogeneity of the prevalence seems pretty robust as consistent through the statistical analyses performed (the subgroup analysis based on the categories of adherence to DSM criteria and the meta-regression with participants’ ages) and with literature on the course of irritability during childhood. Collecting individual-level data would have enabled us to examine the influence of individual factors on DMDD prevalence.

Secondly, the quality of the reviewed information was poor to moderate, especially the definition of DMDD, which widely differed across studies. Only a minority of studies adhered to all criteria. To establish methodological quality, we used a tool based on a subjective assessment of the risk of bias in separate domains relevant to observational studies, such as those recommended elsewhere [45, 61].

Thirdly, publication bias may have influenced our results as we did not conduct a comprehensive search of grey literature. The high LFK index for clinical studies supports a high risk of publication bias that may overestimate the prevalence or the comorbidity rates of DMDD in this group, while data from the community-based samples seemed less prone to publication bias. Besides, inter-rater agreement was only measured for full-text articles assessed for eligibility and not all titles/abstracts. Of note, the selection of articles was more exhaustive here than in the recent meta-analysis by Spoelma, Sicouri [16] on the prevalence of pediatric depressive disorders, where only five articles on DMDD were found.

### Clinical and research implications

Depressive disorder is a leading cause of disability worldwide, accounting for almost 12% of total years lived with disability, with approximately one out of five adolescents experiencing at least one episode of major depression before adulthood [16, 62]. Studies from various settings indicate that an early-onset form is associated with higher severity and worse prognosis than late-onset [63]. Identification and treatment of early childhood-onset forms of depressive disorders represent, therefore, a major challenge.

One of the main criticisms against the validity of DMDD as a distinct psychiatric disorder is related to the lack of specificity of DMDD symptoms, resulting in very high prevalences and questioning the risk of pathologizing normal behavior [14, 26]. Our findings moderate this criticism as the strict use of DSM-5 diagnostic criteria largely lowered the comorbidity rates of DMDD. Therefore, establishing consensus on terminology, definitions, and criteria for DMDD should be an important goal. This will be an important step in facilitating more valid and reliable research. In contrast, considering the high comorbidity rates of DMDD with all forms of studied psychopathology found here, it is difficult to consider DMDD as a specific manifestation of pediatric depression rather than of an anxiety disorder, trauma and stress-related disorder, or a disruptive behavioral disorder.

The lack of studies examining the association between DMDD and neurodevelopmental disorders (except ADHD) is an important shortcoming, considering the interplay between emotional regulation capacities and several developmental domains, such as communication, motor competence, or social cognition [64, 65]. Future studies could examine to which extent individuals with developmental disabilities meeting the criteria for DMDD differed from those without DMDD, as conducted by Pan and Yeh [66] for autistic youths. The relationship between DMDD and trauma-related disorder could deserve more attention, considering that maladaptive parenting strategies have been regarded as a critical mechanism involved in the maintenance of irritability symptoms [2]. Of note, the category of complex post-traumatic stress disorder introduced included in the ICD-11 shares many similarities with DMDD, in particular chronic emotional dysregulation. Considering the relationship between exposure to traumatic experiences and chronic emotional dysregulation in youths [67], the links between the two clinical entities would be worth studying.

Based on existing literature, there is certainly evidence to make a case for developing specific interventions targeting chronic irritability symptoms [27–29]. Such interventions could represent an opportunity to relieve the distress experienced by youths with chronic forms of irritability. Additional research would ultimately help to determine to which extent it could also prevent the risk of developing depressive disorders in adulthood or other forms of psychopathology [31–33, 68–70].

## Supporting information

Benarous et al. supplementary material Benarous et al. supplementary material

## Data Availability

Data are available upon request to the corresponding author.

## Supplementary material

The supplementary material for this article can be found at http://doi.org/10.1192/j.eurpsy.2024.1813.
